# Integral Valorisation of Agri-Food By-Products Through the Production of Food Ingredients Using High-Pressure Thermal Treatments

**DOI:** 10.3390/foods14132214

**Published:** 2025-06-24

**Authors:** Miriam Sánchez-Ordóñez, Jorge A. Saraiva, Carlos A. Pinto, Jonathan Delgado-Adámez, M. Rosario Ramírez-Bernabé

**Affiliations:** 1 Centro de Investigaciones Científicas y Tecnológicas de Extremadura (CICYTEX), Instituto Tecnológico Agroalimentario (INTAEX), 06071 Badajoz, Spain; miriam.sanchezo@juntaex.es (M.S.-O.); jonathan.delgado@juntaex.es (J.D.-A.); 2 Laboratório Associado para a Química Verde/Associated Laboratory for Green Chemistry (LAQV) of the Network of Chemistry and Technology (REQUIMTE), Department of Chemistry, University of Aveiro, 3810-193 Aveiro, Portugal; jorgesaraiva@ua.pt (J.A.S.); carlospinto@ua.pt (C.A.P.)

**Keywords:** agri-food by-products, red pepper, wine pomace, bioactive compounds, food sustainability

## Abstract

This study investigates the production of stable ingredients with high bioactive compound content from agri-food wastes. For the valorization process, high-pressure thermal treatment (HPTT) at different temperatures (65, 75, and 85 °C) at 600 MPa for 5 min was applied to three by-products. These HPTTs were compared with conventional thermal treatments (TTs) carried out at the same temperatures and durations. The by-products studied were red pepper (RP) (*Capsicum annuum*), red wine pomace (RWP) from *Tempranillo*, and white wine pomace (WWP) from *Cayetana*, *Pardina,* and *Montúa*. Winemaking by-products presented higher fiber content compared to RP (RP 1.94%, RWP 38.14%, and WWP 34.46%). In RP, the color parameters such as lightness (L*) and redness (a*) were not significantly affected by HPTT or TT, and the total phenolic content (TPC), total carotenoid content (TCC), and antioxidant activity (ABTS) remained stable with the HPTT. The RWP and WWP were more sensitive to the HPTT, producing important color changes and reducing the bioactive compounds. Color (especially redness) showed positive correlations with TPC and ABTS, which could serve as a predictive indicator. Our study shows that HPTT can significantly improve the valorization of RP and winemaking by-products like pomace, leading to the production of a stable food ingredient characterized by high bioactive compound content.

## 1. Introduction

The circular economy paradigm emphasizes the valorization of food by-products, particularly due to their abundance in bioactive compounds with applications in foods, food additives, and active packaging [1]. Within the European Union, approximately 59 million tons of food waste are generated annually, with fruits and vegetables representing the largest share [2]. Due to their high nutritional content and presence of bioactive constituents, these waste streams represent a promising resource for the development of commercially valuable products [3].

Agro-industrial by-products include grape pomace (red wine pomace, RWP, and white wine pomace, WWP), generated in the winemaking process, and red pepper (RP) waste from varieties that do not meet the size or shape standards for the market as fresh produce. Grape pomaces are composed of skins, seeds, and stems. This by-product is obtained after the wine fermentation, maceration, and decanting processes [4,5] and is rich in dietary fiber and polyphenols, contributing to antioxidant and antimicrobial properties [6]. Similarly, RP (*Capsicum annuum*) by-products are rich in phenols and carotenoids and offer nutritional value and bioactive effects, including antimicrobial, antitumor, and antioxidant properties [7]. Carotenoids such as capsanthin, found in RP, provide numerous health benefits, serving as a source of provitamin A and a natural food colorant [8]. Additionally, RP seeds contain oils rich in polyunsaturated acids and bioactive compounds such as polyphenols, tocopherols, and antioxidants [9,10].

However, due to the seasonal nature of these by-products, they must be stabilized to allow for storage until their eventual use. These by-products often have a high microbiological load and may contain pathogens that can compromise food safety when incorporated into the product. In addition, the vegetable by-products are generally seasonal, and a low microbial load ensures the shelf life of the processed material. Furthermore, the bioactive compounds present in these by-products are often not bioaccessible, which limits their ability to perform a preservative function in food. For these reasons, processing could not only improve safety by inactivating microorganisms but also enhance the bioaccessibility of their active compounds. Previous studies have often focused on using extracts from by-products rather than the whole by-product [11,12,13]. However, extraction methods are often solvent-based, which can pose toxicity risks, lower yields, and increase costs, limiting industrial feasibility.

Recent studies have explored various valorization strategies of agro-industrial by-products. For example, Timón et al. [14] studied the antioxidant capacity of lyophilized extracts of grape, RP, lemon, olive, tomato, and pomegranate by-products. Andrés et al. [15] studied the antioxidant and antimicrobial potential of red grape by-products, which were applied as ingredients in the form of lyophilized extracts to raw lamb patties. Hydrostatic high pressure (HHP) preserved phenolic compounds on RWP and WWP, and reduced the microbial loads after processing, although the polyphenol oxidase enzyme (PPO), responsible for the degradation of phenolic compounds concentration in vegetables, remained active after processing and during the subsequent storage [16,17]. For that reason, HHP required prior blanching in wine pomace to inactivate the PPO and stabilize the phenolic compound content during storage.

High-pressure thermal treatment (HPTT) is an emerging and sustainable technology for preservation in food products. It combines hydrostatic high pressure with moderate heat to produce microbiologically safe ingredients, without the use of solvents, while preserving, while preserving their sensory and nutritional quality [18]. Currently, HPTT is predominantly at the laboratory scale, with only a few pilot-scale units currently in operation worldwide [19]. HPTT can mitigate the negative effects of conventional heat treatment (which exposes foods to long heating and cooling times), thus reducing exposure to high temperatures. Since compression generates heat through adiabatic heating, it rapidly and uniformly increases the temperature of products by approximately 3 °C (in low-fat, more aqueous products) to 9 °C (in higher-fat products) per 100 MPa [20]. This increase in temperature allows sufficient levels to be reached to inactivate bacterial spores in low-acid foods (*Clostridium botulinum*) and enzymes (usually high-pressure resistant) [21]. In this way, microbial stability is improved to the extent that processed foods can be stored at room temperature, reducing energy and storage costs. HPTT is expected to inactivate the PPO since this treatment is more effective than HHP for enzyme inactivation [22]. In addition, HPTT improves the preservation of fresh attributes such as color, aroma, and texture, with much shorter processing times than conventional heat treatments, thanks to the nature of the adiabatic process, as well as fast and uniform heating [23,24]. However, applying HPTT commercially involves several challenges, including the significant capital investment required for equipment and the difficulties associated with scaling up the process while ensuring uniform pressure distribution and consistent product quality. Additionally, higher operational costs, process integration with existing production lines, and regulatory considerations all contribute to the complexity of adopting HPTT technology on a commercial scale.

Regarding bioactive compounds, several authors, such as Gupta et al. [23], have evaluated the impact of HPTT on the retention and bioavailability of carotenoids in matrices like tomatoes and carrots. Garcia-Parra et al. [25] observed that the HPTT of pumpkin puree at moderate temperatures could be an effective alternative to preserve and even increase the bioavailability of health-promoting compounds, such as carotenoids, phenolic acids, and antioxidants. Although several studies have compared HPTT with conventional TT, to our knowledge, its specific application in the valorization of by-products within the agri-food industry has not been evaluated. This technology represents a promising and sustainable alternative, with the potential to reduce agro-industrial waste by transforming by-products into microbiologically stable ingredients enriched in bioactive compounds, thus contributing to a more circular and environmentally friendly model.

The aim of this study was to investigate the use of HPTT for the comprehensive valorization of grape by-products (RWP and WWP) and RP, all of which are rich in bioactive compounds with antioxidant activity. This approach seeks to fully reutilize agri-food waste as safe and bioactive ingredients with a long shelf life, suitable for application in food preservation. The adiabatic heating induced by HPTT inactivates PPO, which may help stabilize phenolics in the treated product during storage, while the intense processing conditions can enhance the safety of the valorized product while preserving the bioactive compounds content.

## 2. Materials and Methods

### 2.1. Raw Materials Preparation and Processing

The RP (*Capsicum annuum*) was obtained from a local company and did not comply with the size required for its commercialization. RWP (var. *Tempranillo*) and WWP obtained from *Vitis vinifera* L. varieties *Cayetana*, *Pardina,* and *Montúa*, traditional varieties grown in the Extremadura region, were provided by a local winery, Santa Marta de los Barros Coop. (Badajoz, Spain). All ingredients were stored at −40 °C until use.

RP (with the seeds and stalk), RWP, and WWP were crushed (individually) at maximum power in a crusher (Thermomix/Bimby, Vorwerk, Carnaxide, Portugal) until a fine and homogeneous product was obtained. Afterwards, 50 g of RP sample and 40 g of pomace (RWP and WWP) were packaged in 12.5 × 12.5 cm heat-sealed bags (low permeability polyamide/polyethylene bags with 90 microns of thickness (PA/PE-90, IdeiaPack—Indústria de Embalagens S.A., Abraveses, Viseu, Portugal). These samples were then subjected to two different processing methods: thermal treatments (TTs) and HPTT.

By-products (RP, RWP, and WWP) were processed under seven different conditions (Table 1). Lot 1 served as control, with no pressure, time, or temperature applied. Lots 2, 3, and 4 were subjected to TT at 65, 75, and 85 °C, respectively, for 5 min. Treatments were carried out in a stainless-steel thermostatic bath (SB series, FALC Instruments, Treviglio, Italy), using distilled water as the heat-transfer medium. Sample temperature was monitored using a K-type thermocouple (Thermometer 305, Roline, Bassersdorf, Switzerland). Once the internal temperature was reached, the samples were kept at the required temperature for 5 min and then rapidly cooled in water (~15 °C).

Lots 5, 6, and 7 underwent HPTT at 600 MPa for 5 min, at 65, 75, and 85 °C. The samples were first preheated in the thermostatic bath to the required temperature and transferred to an insulated basket of polypropylene containing water at the same temperature. The insulated basket (2 L) was sealed with screws and inserted into a 55 L high-pressure equipment (Hiperbaric 55, Hiperbaric S.A., Burgos, Spain), located at the pilot plant of the University of Aveiro. Pressurization occurred at a rate of 3 MPa s^−1^, and depressurization was completed in under 3 s. The choice of pressure parameters was based on previous studies [16,17], which demonstrated by-product stability under refrigeration for at least nine months. The final temperatures during HPTT were estimated considering an adiabatic rise of approximately 3 °C for every 100 MPa applied [20]. From initial temperatures of 65, 75, and 85 °C, final temperatures of 83, 93, and 103 °C were estimated, respectively.

After processing, samples were quickly cooled down for color measurements and subsequently frozen at −80 °C for further analysis. Three heat-sealed bags per product were treated under each condition, resulting in 21 bags for RP, 21 for RWP, and 21 for WWP.

### 2.2. Physico-Chemical Characteristics

Initial by-products were analyzed to evaluate the physico-chemical composition. pH was measured with a pH meter (Hanna Instruments, Eibar, Spain). The water activity (a_w_) measurement was carried out at 25 °C with a Novasina AW-SPRINT-TH 500 instrument (Axair Ltd., Pfäffikon, Switzerland), which gives temperature-controlled measurements. Moisture was determined according to the AOAC [26], and fat content was analyzed by the Folch et al. [27] method. Fiber content was determined by the modified Southgate method [28]. The protein content was determined from the nitrogen content (N × 6.25) by the Kjeldahl method [29].

### 2.3. PPO Activity

The PPO activity was analyzed according to Terefe et al. [30] in all samples. Absorbance was measured at 420 nm and 25 °C for 3 min in a Thermo Scientific Evolution 201 UV-Vis spectrophotometer (Thermo Scientific™, Fisher Scientific SL, Madrid, Spain), in kinetic mode. The enzyme’s activity was quantified as a percentage relative to the activity observed in the control samples.

No activity was found for this enzyme in RP, RWP, and WWP sample controls, TT, and HPTT.

### 2.4. Instrumental Color

The color was evaluated using the Konica Minolta CM 2300d spectrophotometer colorimeter (Konica Minolta Inc., Tokyo, Japan). Once calibration was completed, measurements CIE L* (representing lightness), a* (indicating redness/greenness), and b* (indicating yellowness/blueness) were taken. Color measurements were made directly on the sample bag, taking care not to get bubbles, without removing the product so that it would not oxidize. The same sample was measured in triplicate, each one measured six times in different positions.

The total color differences parameter (ΔE) was calculated to evaluate differences between samples before and after processing. The following equation was used:

$$\Delta E=\sqrt{{\left(L^{*}-L_{0}^{*}\right)}^{2}+{\left(a^{*}-a_{0}^{*}\right)}^{2}+{\left(b^{*}-b_{0}^{*}\right)}^{2}}$$

where L*_0_, a*_0_, b*_0_ are the control values for unprocessed products and L*, a*, b* are the parameters of samples after HPTT and TT. According to the values of the parameter, the differences could be estimated as not noticeable (0 to 0.5), slightly noticeable (0.5 to 1.5), noticeable (1.5 to 3.0), well visible (3.0 to 6.0), and great (6.0 to 12.0) [31].

### 2.5. Total Phenolic Compounds (TPC)

To carry out the extraction, 5 g of sample control obtained by TT and HPTT were added to 45 mL of solution (water 80%, 19.9% methanol, 0.1% citric acid). The mixture was agitated for 1 h at 60 °C and centrifuged at 1000 rpm for 5 min at 4 °C. The supernatant was evaporated at 37 °C for 20 min in a rotary evaporator at 50 mbar of vacuum. Finally, the solvent-free extract was made up to a volume of 50 mL with ultra-pure water. A sample extract (1 mL) was analyzed by a protocol based on the Folin–Ciocalteu colorimetric method described by Lima et al. [32]. The results were expressed in mg gallic acid equivalent (GAE) on 100 g of wet sample by the utilization of a calibration curve with gallic acid.

### 2.6. Total Carotenoid Content (TCC)

TCC was extracted following the method described by Bohoyo-Gil et al. [33] with some modifications: 5 g of fresh sample was weighed and washed four times with acetone (10 mL for the first three and 15 mL for the fourth). The mixture was subjected to ultrasound for 10 min, then centrifuged at 10,000 rpm for 2 min at 4 °C. The supernatant was filtered in Whatman Nº 1 paper and made up to 50 mL in an amber volumetric flask with acetone. A filtered aliquot (0.45 µm) was diluted 1:5 in acetone, and the absorbance was measured in a Thermo Scientific Evolution 201 UV-Vis spectrophotometer (Thermo Scientific™, Fisher Scientific SL, Madrid, Spain) between 300 and 500 nm. The total carotenoid content was expressed as µg g^−1^ of the wet sample.

### 2.7. ABTS Method (2,2′-Azino-bis (3-ethylbenzothiazoline) 6-sulfonicacid)

The total antioxidant activity was determined by the ABTS^+^ spectrophotometric method [34]. The absorbance readings were carried out on a plate reader (Tecan, spark multimode microplate reader model, Grödig, Austria). The method consists of obtaining a blue/green chromophore ABTS^+^ through the oxidation of ABTS with potassium persulphate. The presence of antioxidants reverses the oxidized ABTS^+^ radical to ABTS, resulting in a reduction in the blue/green color that gives place to an absorption reduction measured at 734 nm. ABTS radical solution was prepared by mixing 7 mM ABTS and 2 mM potassium persulphate. The solution was combined with phosphate-buffer saline (PBS) and stored in the dark for 12–16 h at 4 °C to obtain an absorbance of 1.32 at 734 nm. Finally, an aliquot of extract was added to the ABTS^+^ solution, and the decrease in absorbance at 734 nm was measured. Trolox was used as a reference compound. The results were expressed as μM Trolox Equivalent (TE) per ml extract.

### 2.8. Statistical Analysis

All results are presented as mean ± standard deviation for each designed group. One-way analysis of variance (ANOVA) (*p* ≤ 0.05) was applied to compare the effect of processing. A Tukey-b test was applied to compare the mean values when ANOVA showed significant differences. Pearson correlation analysis was performed to evaluate the relationships between color parameters (L*, a*, b*), ABTS, and TPC. The significance levels were set at *p* ≤ 0.05 and *p* ≤ 0.01 for statistical relevance, and the software SPSS, Version 21.0 (SPSS Inc., Chicago, IL, USA) was utilized.

## 3. Results and Discussion

### 3.1. Chemical Composition of the By-Product

Table 2 shows the chemical composition of RP, RWP, and WWP. Significant differences in the pH were found among the by-products analyzed. RP showed the highest values, followed by RWP, and finally, WWP presented the lowest values. According to the pH and its effect on microbial development, RP could be considered a low-acid food (pH > 4.6), while both types of pomaces presented high acid pH (pH < 4.6). The a_w_ and moisture content were significantly higher in RP than in both pomaces, with RWP showing intermediate values. The main difference between by-products lies in their origin: in the case of RP, it is the whole fruit that does not reach a commercial size, while pomace comes from the solid residue left over after the juice or must have been extracted in winemaking. This distinction is further accentuated by the differences between RWP and WWP. In the traditional white winemaking process, the seeds and skins are removed before fermentation, with only the juice (must) undergoing fermentation [4,35], so WWP usually has more sugars, more water, and no alcohol compared to RWP.

Several authors have investigated the chemical composition of these products. For instance, Castro et al. [36,37] have reported in RP ranges of pH between 4.81 and 4.95 and ranges of moisture between 92.0 and 92.8 g 100 g^−1^. In RWP, the values of pH, a_w,_ and moisture were lower than those reported by D’Arrigo et al. [16], despite both were from the same variety, and even samples were taken from the same winery, but in different years. These differences might be due to acidification caused by microbial proliferation during the exposure period before collection. With respect to WWP, Ramírez et al. [17] reported that WWP from the same variety presented similar pH (3.66) but higher values of a_w_ (0.97) and moisture (56.7%) values than our study, probably due to weather conditions in production or the intensity of pressing in each winery. The physical–chemical composition and the content of bioactive compounds of pomace depend on the temperature and time of dehydration [38,39] as well as on the grape variety, climatic conditions, and cultivation practices.

Significant differences in fat, fiber, and protein content were found between RP and pomace (RWP and WWP). RP showed the lowest levels of the three components analyzed. Despite its low-fat content, RP contains carotenoids, lipophilic compounds whose distribution is linked to the lipid profile of plants [40]. The carotenoids may be present in the seeds, which, although small in quantity, are rich in polyunsaturated fatty acids [9,10,41].

RWP had the highest fat, fiber, and protein content, while WWP showed intermediate values. Overall, grape pomace is a good source of seeds, which explains its richness in dietary fiber and polyunsaturated fatty acids, both of which have beneficial health effects, such as facilitating the transport of antioxidants in the digestive tract [42,43]. The fat, fiber, and protein composition of RWP is in agreement with the findings of D’Arrigo et al. [16] who studied an RWP of the same variety and with Ramírez et al. [17] in WWP. Antonić et al. [44] also showed fat values within the range (1.14–13.90%) in the review about different varieties of red and WWP chemical composition (dry weight).

### 3.2. Instrumental Color, Color Changes, Nutritional Molecules, and Antioxidant Activity of Procesed By-Products

The instrumental color parameters, bioactive compounds content, and their bioactivity (antioxidant effect, ABTS) of the RP (Table 3), the RWP (Table 4), and the WWP (Table 5) subjected to different TT and HPTT were evaluated, and compared to non-treated samples (control).

#### 3.2.1. RP (*Capsicum annuum*)

The lightness (CIE L*) of RP was not affected by the application of TT or HPTT. Similarly, the redness (CIE a*) was not modified after processing (TT or HPTT). However, the yellowness (CIE b*) was significantly reduced in all treatments (TT and HPTT). The HPTT conditions at 65 and 75 °C considerably reduced the yellowness of the samples compared to the control, while the other treatments maintained intermediate values compared to the initial values (control). The color parameters were different compared to those reported by Woldemariam et al. [45] in RP paste after HHP treatments (100–600 MPa/30–600 s) with values of L*, a* and b* ranging from 29.89 to 31.83, 12.62 to 14.12 and 5.88 to 8.89, respectively. Differences could be attributed to the lower moisture content of the pepper paste compared to the whole fresh pepper used in the current assay.

The vibrant red color of RP is due to several carotenoid pigments, such as β-carotene, which has pro-vitamin A activity, and oxygenated carotenoids such as capsanthin, capsorubin, and cryptocapsin, which are characteristic of this genus and known for their efficiency in neutralizing free radicals [46]. In our study, it was observed that the HPTT and TT conditions did not degrade the red color of the by-products, suggesting that certain red pigments, such as capsanthin and capsorubin, could be preserved under these processing conditions. Woldemariam et al. [45] reported that HHP (100–600 MPa/30–600 s) did not affect the levels of carotenoids in RP paste. However, these pigments can be sensitive to high temperatures, as observed by Mengistu and Beri [47] in their review of the impact of thermal processing on RP (*C. annuum*). In this regard, studies on the effect of thermal drying treatment (60 °C, 200–1000 min) on RP have reported a decrease in the parameters L*, a*, and b* [48], which could be attributed to processing conditions that were more intense than in the current experiment. This is the first study that evaluates the effect of HPTT on RP, although the effects of TT treatment or the dehydration process to obtain paste or dried paprika have been extensively studied [47]. In this sense, color changes depend on the processing, type of heat transfer, temperature, and time [49]. This suggests that HPTT may be a processing method that retains effectively the characteristic colors of RP.

The global color changes (ΔE) of RP were calculated to evaluate the effect of processing in all instrumental color parameters, and they were significantly modified at all processing conditions (TT and HPTT). According to the values of the parameter (ΔE), the changes could be estimated as not noticeable (0 to 0.5), slightly noticeable (0.5 to 1.5), noticeable (1.5 to 3.0), well visible (3.0 to 6.0), and great (6.0 to 12.0). The HPTT at 65 and 75 °C showed the most intense changes, with values of 7.48 and 7.80, categorized as “great” color changes. The treatments TT (65 and 75 °C) and HPTT (85 °C) had the lowest values, between 3.16 and 4.69. These changes could also be classified as “well visible”. It is interesting to note that the most intense HPTT conditions (600 MPa/85 °C) applied showed similar modifications to the less intense TT, so considering color changes, this HPTT would be the most appropriate.

García-Parra et al. [25] also observed that HPTT (300, 600, 900 MPa/60, 70, 80 °C/1 min) in pumpkin puree (a matrix rich in carotenes like RP. Specifically, the ΔE values after processing ranged from 3 to 4 (classified as “well visible” changes), being lower than the treatments of HPTT at 65 or 75 °C in this study. However, in RP paste treated with different HHP (100–600 MPa/30–600 s) [45], color changes (ΔE) values were less intense and ranged between 0.2 and 2.8. They were classified as “not noticeable” to “slightly noticeable” or “noticeable”. The lower values of color changes (ΔE) in the pumpkin after HPTT than in the current study could be explained by differences in holding times of the HPTT (1 min vs. 5 min), while the less intense effect of HHP on the color changes in RP paste could be caused by the lack of heating in the treatments applied (HHP).

TPC of RP (Table 3) in TT at 65 and 85 °C, together with all the HPTT, retained the initial levels. However, TPC was significantly affected after TT at 75 °C, reducing the 37% with respect to the control, probably due to the thermal sensitivity of the polyphenolic compounds and the breaking of bonds with the cell wall which facilitates their degradation [47].

**Table 3:** Instrumental color parameters, color changes (ΔE: control vs. treated), TCC (μg g^−1^ wet sample), TPC (mg GAE 100 g^−1^ wet sample), and ABTS (μM TE mL^−1^ extract) of RP with different treatments.

	Control	TT (5 min)			HPTT (600 MPa/5 min)			*p*
		65 °C	75 °C	85 °C	65 °C	75 °C	85 °C	
**Instrumental color**								
CIE L*	43.72 ± 1.51	41.95 ± 0.81	41.89 ± 0.80	42.29 ± 1.02	40.68 ± 0.44	41.13 ± 0.42	41.53 ± 1.81	ns
CIE a*	41.03 ± 1.91	40.97 ± 0.40	39.81 ± 0.39	40.01 ± 0.41	39.46 ± 0.38	39.64 ± 0.55	40.82 ± 2.15	ns
CIE b*	52.22 a ± 0.32	48.29 b ± 1.42	45.20c ± 1.12	42.96 cd ± 0.76	41.28 d ± 1.89	40.68 d ± 0.45	48.20 b ± 0.54	***
**Color changes**								
ΔE	-	4.69 b ± 1.46	3.83 b ± 0.58	5.19 ab ± 0.71	7.48 a ± 1.89	7.80 a ± 0.46	3.16 b ± 0.78	**
**Nutritional molecules**								
TPC	106.40 a ± 9.60	98.14 a ± 6.33	66.76 b ± 0.73	95.14 a ± 6.14	95.80 a ± 10.43	92.58 a ± 6.27	100.44 a ± 8.41	***
TCC	146.52 ± 10.52	140.15 ± 8.73	132.43 ± 6.14	157.18 ± 21.01	153.31 ± 6.06	160.56 ± 17.76	139.72 ± 13.07	ns
**Antioxidant activity**								
ABTS	1.04 ± 0.01	0.90 ± 0.05	1.01 ± 0.06	0.94 ± 0.05	0.94 ± 0.06	0.99 ± 0.05	0.95 ± 0.05	ns

Values represent mean ± standard deviation. Different letters in the same row indicate statistical differences (Tukey-b’s test). ** *p* < 0.01, *** *p* < 0.001. ns: Non-significant differences, *p* > 0.05.

TCC in control was 146.5 µg g^−1^ in fresh weight, which corresponds to approximately 1.7 g 100 g^−1^ on a dry weight basis, a value that falls within the range reported in the literature (0.1–3.2 g 100 g^−1^ dry weight). However, notable variability has been observed depending on cultivars, processing methods, and growing conditions [40]. This aspect is particularly relevant as using the whole product as a by-product, the nutritional benefits of the complete fruit are utilized. TT and HPTT did not significantly modify the TCC with respect to RP control. Oxidation can easily degrade carotenoids due to their structure with multiple conjugated double bonds. In addition, heat treatment can cause isomerization of β-carotene to cis forms or its fragmentation, which significantly reduces its provitamin activity [50,51]. In our study, no changes in TCC were observed after TT and HPTT. This suggests that treatments do not induce isomerization or oxidation. The observed stability of TCC may be attributed to the resistance of carotenoid compounds to the thermal treatment [52]. The slight increases in carotenoids (although changes were not significant) could be attributed to the cell wall rupture caused by high pressure, which facilitates the release and accessibility of intracellular compounds. Probably at more intense conditions than those applied in our experiments, the increases could be more evident. In line with our results, García-Parra et al. [25] found that HPTT at 60, 70, 80 °C and 300, 600, and 900 MPa effectively maintained or increased the carotenoid content of pumpkin puree. Intense HPTT may even increase carotenoid extractability due to pressure-induced structural changes in cell membranes [25]. Sánchez et al. [53] showed that the carotenoid content of commonly consumed vegetables (carrot, tomato, or red bell pepper) was not significantly influenced by HHP treatment (625 MPa, 5 min, 20 °C) or HPTT (625 MPa, 5 min, 70 °C and 625 MPa, 5 min, 117 °C).

ABTS remained stable after all treatments, indicating that none of them caused a decrease in the antioxidants present in the initial samples, maintaining the initial antioxidant capacity throughout the process. ABTS and TCC are strongly related because carotenoids act as antioxidants, deactivating free radicals and quenching reactive oxygen species due to the presence of conjugated double bonds [54]. This would corroborate similar effects after the TT and HPTT. Moreover, studies have demonstrated the high antioxidant capacity of RP pericarp and seed extracts [55]. In general, *Capsicum* cultivars have been recognized as vegetables with remarkable antioxidant potential [56]. RP of the species *C. annuum* present phenolic compounds (hydroxybenzoic and hydroxycinnamic acids), flavonoids such as luteolin and catechin [57] or apigenin, luteolin, quercetin, rutin, galangal, naringenin [58] and glycoside derivatives [59,60,61]. The antioxidant activity is attributed not only to phenolic compounds, but also to flavonoids, carotenoids, and tocopherols [7]. Therefore, as TPC and antioxidant activity were not affected by HPTT, other bioactive compounds with antioxidant activity would have been well preserved after processing.

Studies on other vegetables have indicated that HHP at 600 MPa preserved the antioxidant activity (DPPH) of tomato juice [62] and ABTS of the tomato–carrot mixture [50]. Indrawati et al. [63] also observed an increase in antioxidant activity (ABTS) when processing carrot juice under pressures of 100 to 800 MPa, temperatures of 30 to 65 °C, and a maximum duration of 90 min.

Overall, HPTT presented a similar effectiveness as the TT applied in preserving the initial color in CIE L* and CIE a* (related to carotenoid stability), TPC, TCC, and bioactivity in RP. In addition, although the color changes were more noticeable with HPTT at 65 and 75 °C, they did not compromise the quality attributes of the samples. Additionally, it should be noted that the HPTT conditions starting at initial temperatures of 65, 75, and 85 °C may have resulted in final temperatures of approximately 83, 93, and 103 °C, respectively. Therefore, in all cases, the HPTT was more intense than the TT due to the adiabatic heating. In this case, the microbial inactivation would be more effective in the HPTT than in the TT, and thus, a longer shelf-life of the valorized by-products would be expected. Studies about HPTT in pumpkin (rich in carotenoids) demonstrated the effectiveness of this type of treatment (60, 70, 80 °C and 300, 600, and 900 MPa) on microorganisms [25]. Considering the lower pH of red pepper than pumpkin, the effectiveness of those conditions (equivalent to ours) would be even greater from a microbiological point of view. This would support the advantage of using HPTT at 85 °C over TT. HPTT could be used to valorize RP by-products, extending their shelf life and facilitating their incorporation into foods as a natural source of phenols and antioxidants.

#### 3.2.2. RWP (*Tempranillo*)

The instrumental color (Table 4) of RWP was (L* 34.5; a* 5.8; b* 3.6). Compared to previous studies, D’Arrigo et al. [16] reported differences in redness and yellowness in RWP of the same variety (var. *Tempranillo*) (L* 36.3–36.4; a* 4.0; b* 0.4). Similarly, results from Xu et al. [64] showed differences concerning our study in both L*, a*, and b* in RWP skin. Differences could be attributed to seasonal differences in grapes or even to the grade of dehydration of the pomace, which is generally stored in the sun to lose weight.

**Table 4:** Instrumental color parameters, color changes (ΔE: control vs. treated), TPC (mg GAE 100 g^−1^ wet sample), and ABTS (μM TE ml^−1^ extract) of RWP with different treatments.

	Control	TT (5 min)			HPTT (600 MPa/5 min)			*p*
		65 °C	75 °C	85 °C	65 °C	75 °C	85 °C	
**Instrumental color**								
CIE L*	34.54 a ±1.68	33.62 ab ± 0.79	33.29 ab ± 0.33	32.60 ab ± 0.48	34.65 a ± 0.10	31.82 b ± 0.24	34.22 a ± 0.27	ns
CIE a*	5.85 a ± 0.23	6.77 a ± 0.32	6.34 a ± 0.53	6.32 a ± 0.11	4.45 b ± 0.42	4.19 b ± 0.36	4.34 b ± 0.37	ns
CIE b*	3.58 b ± 0.26	4.89 a ± 0.36	4.63 a ± 0.25	4.65 a ± 0.08	3.19 ab ± 0.12	3.35 ab ± 0.25	2.95 b ± 0.05	***
**Color changes**								
ΔE	-	1.91 ab ± 0.72	1.75 b ± 0.45	2.27 ab ± 0.46	1.46 b ± 0.41	3.20 a ± 0.36	1.68 b ± 0.37	**
**Nutritional molecules**								
TPC	107.78 a ± 1.32	93.98 abc ± 4.79	95.90 ab ± 4.30	79.62 cd ± 6.93	72.05 d ± 10.68	66.44d ± 1.01	81.51bcd ± 0.46	***
**Antioxidant activity**								
ABTS	1.30a ± 0.01	1.18ab ± 0.03	1.20a ± 0.04	1.05b ± 0.07	0.91c ± 0.09	0.90c ± 0.02	1.06b ± 0.03	ns

Values represent mean ± standard deviation. Different letters in the same row indicate statistical differences (Tukey-b’s test). ** *p* < 0.01 *** *p* < 0.001. ns: Non-significant differences, *p* > 0.05.

The instrumental color of RWP was significantly modified after the treatments were applied. The lightness (CIE L*) of the RWP remained stable at HPTT 65 and 85 °C compared to the control (unprocessed), but the HPTT 75 °C reduced the L* values. The TT showed intermediate values compared to the control. Regarding the redness (CIE a*), all TT groups maintained stable a* values compared to the control, while all HPTT reduced the redness. Red color reductions could be caused because the compounds responsible for the coloring in RWP, such as anthocyanins, can be sensitive to HPTT. Yellowness (CIE b*) increased with all TT, but HPTT at 85 °C maintained stable values compared to the control, while the other HPTT (65 and 75 °C) showed intermediate values.

ΔE was used to evaluate global color changes after processing, and it showed significant differences among treatments. The lowest color changes were found after TT at 75 °C, HPTT at 65 °C, and HPTT at 85 °C (ΔE 1.46–1.75), which could be considered “slightly noticeable” to “noticeable” to the human eye. Despite the reduction in CIE a* after all HPT conditions, global changes in RWP after HPTT at 65 °C and 85 °C were not easily perceived by the human eye. These treatments preserved best the original red-brownish color of the pomace. In contrast, HPTT at 75 °C resulted in the most noticeable color changes (ΔE 3.20), which could be classified as “well visible,” suggesting that this treatment should be avoided.

Jesus et al. [65] found that HPTT at 600 MPa/65 °C for 15 min preserved the red color of açai pulp, with an ΔE value of 1.13, which could be considered a “slightly noticeable” color change after processing. The effect of HPTT has not been previously evaluated on grape pomace, although some studies have evaluated the effect of HHP. D’Arrigo et al. [16] reported no color changes in the *Tempranillo* RWP after HHP (400–600 MPa/1–300 s/1 or 2 cycles). The parameter ΔE processing ranged between 0.3 and 0.8, so changes after HHP were considered “not noticeable” in that study. However, a significant discoloration occurred during storage, likely due to the active PPO, which remained unaffected by HHP. However, in our study, surprisingly no PPO activity was found in the unprocessed pomace, so that color could presumably be well preserved after processing. The lack of activity of this enzyme in the current pomace was unexpected, and we consider that environmental factors, and the manipulation in the industry (inactivation during processing, storage conditions) of the RWP could explain these results. PPO is an enzyme that may be present in grape pomace [66] and is directly involved in the degradation of phenolic compounds [67]. PPO catalyzes the hydroxylation of monophenols to o-diphenols, followed by their oxidation to o-quinones, which subsequently polymerize to form undesirable dark-colored pigments [68]. An increase in temperature can lead to a reduction in PPO activity. For example, studies on *Pinot Noir* grape pomace have shown that drying at 60 °C helps preserve phenolic compounds more effectively [69]. This suggests that drying at moderate temperatures may inhibit PPO activity, thereby minimizing the degradation of phenolics. We hypothesize that while the complete absence of PPO activity in pomace is uncommon, certain processing methods, such as drying at higher temperatures, may significantly reduce its activity. In this case, the pomace was exposed to sunlight for several days before being taken for this experiment. This practice, commonly employed by wine producers, aims to reduce the moisture content and overall weight of the pomace prior to transportation. Other studies carried out in the same winery showed a differential activity, probably because the pomace was fresher.

Phenolic compounds in RWP were significantly modified following all treatments. HPTT at 65 °C and 75 °C resulted in the lowest TPC, with reductions of approximately 33% and 38%, respectively, compared to the initial concentration. The rest of the treatments (all TT and HPTT/85 °C) showed intermediate results with ranges between the previous and the control samples. TT at the lower temperatures of 65 °C and 75 °C best preserved the initial levels of TPC. Similarly, the antioxidant activity presented significant differences after processing. TT 75 °C maintained the antioxidant activity with respect to the control. However, HPTT at 65 and 75 °C caused a significant reduction in the antioxidant activity, while other treatments presented intermediate decreases. The reduction in phenolic compounds and antioxidant activity after processing may be attributed to the polymerization of polyphenols at high temperatures, which reduces the number of active hydroxyl groups available to scavenge free radicals [70,71]. It should also be considered that during HPTT the temperature increase is caused by adiabatic heating, with estimated final temperatures of 83, 93, and 103 °C when starting from 65, 75, and 85 °C, respectively [20]. These elevated temperatures may have contributed to the thermal degradation or transformation of phenolic compounds, further affecting their antioxidant potential.

D’Arrigo et al. [16] reported that the phenolic compounds of RWP remained stable after HHP at 600 MPa/300 s/16 °C. Sánchez-Ordóñez et al. observed that phenolic compounds in RWP increased slightly after thermal scalding (103 °C for 1 min) and that subsequent HHP (600 MPa, 5 min, at 16 °C) was effective in preserving these compounds. However, our results were different, as phenolic compounds decreased significantly with HPTT due to adiabatic heating, which caused a temperature increase from the applied pressure. Differences in the behavior of TPC on RWP in that study compared to this one could be attributed to the way of application of the thermal blanching, which was applied in the whole pomace (non-milled product) so that the internal temperature of the pomace probably could not reach the applied temperature during the scalding. On the other hand, Corrales et al. [72] observed higher antioxidant capacity in red grape skin extracts at 600 MPa/70 °C/60 min, suggesting an effective antioxidant extraction under those conditions. Differences in the methods, such as the use of solvents in their study and the absence of solvents in ours, may explain the variation in results.

In general, TT preserves color better and generates less visual disturbance compared to HPTT. In addition, TT has a milder impact on phenols and maintains total antioxidant activity compared to HPTT. RWP was more sensitive to HPTT than to TT, probably due to adiabatic heating, which increases the final temperature of the treatment. This could affect the phenolic compounds responsible for its characteristic color, such as anthocyanins, which could be affected by the temperature increase. However, given its low pH (3.48, Table 2), HPTT may still offer advantages in pathogen inactivation [21], contributing to the microbial safety of the product, and increasing the shelf-life of the treated product.

#### 3.2.3. WWP (*Cayetana*, *Pardina*, *Montúa*)

WWP presented values of instrumental color (L* = 38.9, a* = 8.8, b* = 12.4) (Table 5) that slightly differ from other studies. Ramírez et al. [17] reported instrumental color parameters in WWP from the same varieties with higher values of lightness, lower redness, and similar yellowness (L* = 44.9, a* = 7.6, b* = 12.3) compared to the current study, corresponding to a reddish-brown visual color in this WWP. Since the grape variety and the winery were the same, differences could be mostly attributed to the year.

**Table 5:** Instrumental color parameters, color changes (ΔE: control vs. treated), TPC (mg GAE 100 g^−1^ wet sample), and ABTS (μM TE ml^−1^ extract) of WWP with different treatments.

	Control	TT (5 min)			HPTT (600 MPa/5 min)			*p*
		65 °C	75 °C	85 °C	65 °C	75 °C	85 °C	
**Instrumental color**								
CIE L*	38.88 abc ± 0.61	39.66 a ± 0.15	38.33 bcd ± 0.44	38.78 abc ± 0.18	39.19 ab ± 0.08	37.70 d ± 0.24	38.12 cd ± 0.18	ns
CIE a*	8.78 a ± 0.19	8.78 a ± 0.08	8.62 a ± 0.06	8.39 a ± 0.34	6.49 b ± 0.37	7.20 b ± 0.23	7.34 b ± 0.51	ns
CIE b*	12.40 a ± 0.33	12.92 a ± 0.03	11.84 ab ± 0.09	11.16 b ± 0.07	8.37 b ± 0.17	8.60 b ± 0.27	9.14 b ± 0.67	***
**Color changes**								
ΔE	-	0.94 b ± 0.11	0.82 b ± 0.37	1.32 b ± 0.18	4.66 a ± 0.11	4.29 a ± 0.3	3.66 a ± 0.78	**
**Nutritional molecules**								
TPC	205.59 a ± 1.95	200.77 ab ± 5.58	171.91 cd ± 11.84	186.26 bc ± 0.32	176.57 c ± 1.78	155.07 de ± 7.88	151.39 e ± 1.13	***
**Antioxidant activity**								
ABTS	2.39 a ± 0.07	2.30 abc ± 0.01	2.13 bc ± 0.19	2.36 ab ± 0.1	2.07 cd ± 0.03	1.83 de ± 0.04	1.78 e ± 0.05	ns

Values represent mean ± standard deviation. Different letters in the same row indicate statistical differences (Tukey-b’s test). ** *p* < 0.01 *** *p* < 0.001. ns: Non-significant differences, *p* > 0.05.

The instrumental color of WWP was significantly affected after TT and HPTT. The highest values of lightness (CIE L*) were obtained after the TT at 65 °C while the lowest L* value was obtained in HPTT at 75 °C. The other treatments resulted in intermediate L* values, ranging from 38.12 to 39.19. The CIE a* remained stable after all TT. In contrast, HPTT produced a decrease in a* values (6.49–7.34) at all processing conditions, indicating a significant reduction in the red color intensity of the WWP. With respect to yellowness (CIE b*), TT at 65 °C preserved values, and TT at 75 °C exhibited intermediate values. With the increase in the temperature (TT at 85 °C, HPTT at 65–75–85 °C), a significant reduction in yellowness was observed. WWP is rich in flavonols [73], which provide a golden or yellowish tone and can be affected by the complex interaction between heat and pressure.

Few studies have evaluated the instrumental color of pomace, although Ramírez et al. [17] analyzed the preservation of WWP by HHP (400, 600 MPa/1, 6 min/16 °C) and observed that the different treatments did not affect the color parameters a* and b*. This suggests that the temperature of the high-pressure treatments is crucial for changes in the WWP pigments.

Global color changes (ΔE) presented significant differences among treatments. WWP color changes after TT showed the lowest values ranging from 0.5 to 1.5, classified as “slightly noticeable”. After all HPTT of WWP, global color changes were higher and considered “well visible” with values between 3.66 and 4.66. This is associated with the significant reduction shown in the L*, a*, and b* values compared to the control WWP. In general, the TT conditions would maintain better than HPTT the visual characteristics of the WWP. HHP treatment at 600 MPa/6 min/16 °C produced “slightly noticeable” color changes (ΔE 1.4) in WWP [17]. The effect produced by HHP treatment in that study was similar to that found for the TT, but lower than HPTT, which could be associated with the effect of the temperature of processing (higher in HPTT than in TT).

Changes after HPTT were higher in WWP than in RWP, while TT minimally affected both pomaces. Differences in the phenolic profile of WWP and RWP could be behind these differences. So, WWP presented high levels of phenolic compounds such as flavanols [73], while the phenolic profile of RWP also presented a high level of flavonols and anthocyanins see differences in the amount and type of predominant phenolic compounds could induce different reactions in the color change when the products are subjected to high pressure together with heat treatment (HPTT).

Untreated WWP had 205.59 mg 100 g^−1^ of TPC, which can provide antioxidant properties (2.39 µml ml^−1^). The TPC and the antioxidant activity were reduced as the temperature was increased; the effect was more intense in the HPTT than in the TT. The lowest values in TPC were found with the HPTT at 85 °C, with reductions of 26% for phenolic compounds content and the lowest ABTS with respect to the control pomace. The TT at 65 °C was the best preserved the TPC and the antioxidant activity. This finding is of great interest, as it could be an innovative strategy to obtain WWP rich in bioactive compounds, which would facilitate its conservation and incorporation in various products [17,73]. Previous studies have reported that HHP (600 MPa/6 min/16 °C) [17] or thermal blanching (103 °C for 1 min) [73] did not affect the TPC of WWP. Therefore, TPC would be well preserved by HHP; however, for the application of HPTT, maybe shorter holding times than those applied in this study would be required.

In general, WWP subjected to HPTT exhibited more pronounced color changes compared to TT. Regarding TPC and antioxidant activity, the effect of HPTT at 65 °C was generally similar to that of TT. Nevertheless, under more severe conditions (HPTT at 85 °C), WWP exhibited increased sensitivity, possibly attributed to the additional effect of adiabatic heating, which degraded the bioactive compounds, mostly phenolic compounds. It is important to highlight that WWP exhibits a low pH (3.31), a condition under which HPTT has been reported to be particularly effective in inactivating pathogenic microorganisms [21] and obtaining a safe by-product.

### 3.3. Global Effect of Processing on the By-Products

**Figure 1:**
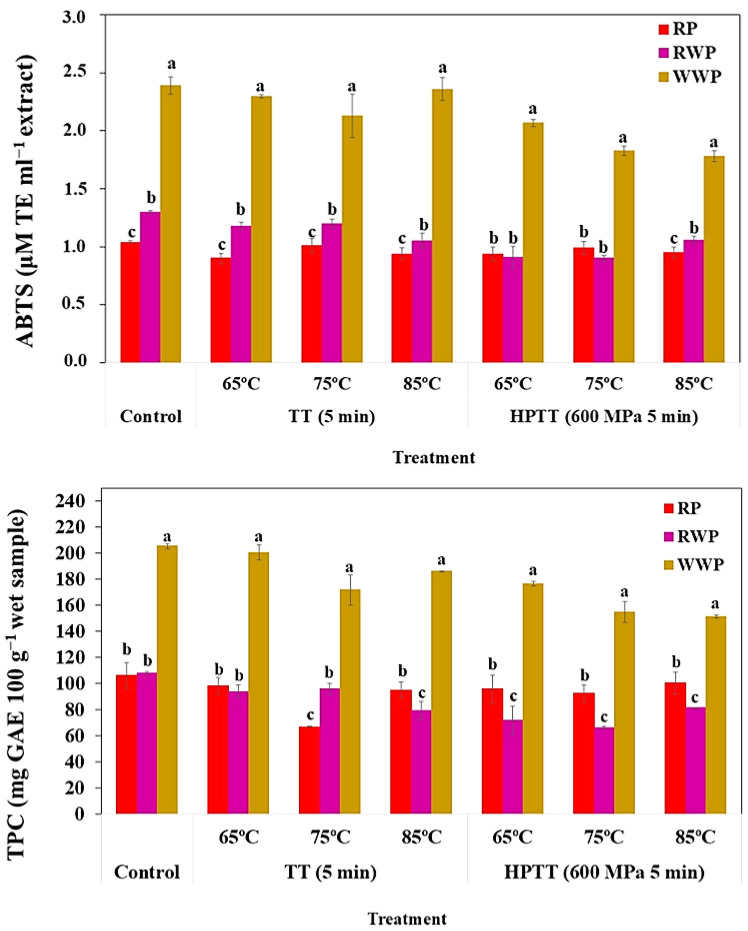
Total phenolic compound content (TPC) and antioxidant activity (ABTS) in red pepper (RP), red wine pomace (RWP), and white wine pomace (WWP) subjected to heat treatments (TT, at 65, 75, and 85 °C for 5 min) and high-pressure thermal treatment (HPTT at 65, 75 and 85 °C for 600 MPa and 5 min). Values represent mean ± standard deviation. Different letters indicate statistical differences (Tukey-b’s test. *p* < 0.05) among the three by-products treated at the same processing conditions.

TPC and antioxidant activity (ABTS) (Figure 1) showed significant differences (*p* < 0.05) among RP, RWP, and WWP treated at the same processing conditions. WWP exhibited the highest values (TPC: 205.59–151.39 mg 100 g^−1^; antioxidant activity: 2.39–1.78 µmol mL^−1^), followed by RWP (TPC: 107.78–66.44 mg 100 g^−1^; antioxidant activity: 1.30–0.90 µmol mL^−1^), and RP (TPC: 106.40–66.76 mg 100 g^−1^; antioxidant activity: 1.04–0.90 µmol mL^−1^). The elevated TPC and antioxidant activity in WWP could be attributed to its composition and the proportion of stalks, skins, seeds, and pulp from unfermented grapes, which are rich in phenolic compounds. In contrast, RWP is obtained after fermentation, a process that can reduce phenolic concentration due to the transmission to the juice. RP presented similar initial values of TPC as RWP, and both were lower than WWP. However, ABTS in RP was lower than WWP and RWP, which presented intermediate levels. RP is characterized by its high content of carotenoid compounds that confer significant antioxidant activity; however, due to their higher moisture content compared to pomace, their relative effect could be diluted.

Interestingly, both TPC and ABTS were better preserved in RP than in grape pomace (RWP and WWP) after HPTT. This suggests a greater resistance of carotenoid-rich matrices to thermal degradation [25,74]. In contrast, the antioxidant activity in grape pomace, primarily dependent on phenolic compounds, was more affected, likely due to their heat sensitivity.

These results differ from Woldemariam et al. [45], who found higher TPC and antioxidant activity in RP, likely due to the low moisture content of the raw material (the RP paste was made with dried peppers). On the other hand, although RWP is often reported to have more phenolics than WWP [16], several studies support high values in WWP [17,75,76]. In part, the lower levels of phenolic compounds in RWP may be due to the extraction of many of these compounds during the fermentation and maceration processes with wine. Additionally, variations in pomace composition could contribute to these differences. The higher moisture content in RWP compared to WWP may dilute its concentration of bioactive compounds (Table 1). Furthermore, the greater fiber content in RWP (Table 1) might be explained by a higher proportion of stems and seeds, which can influence the overall phenolic composition of the final mixture.

### 3.4. Correlation Analysis

**Figure 2:**
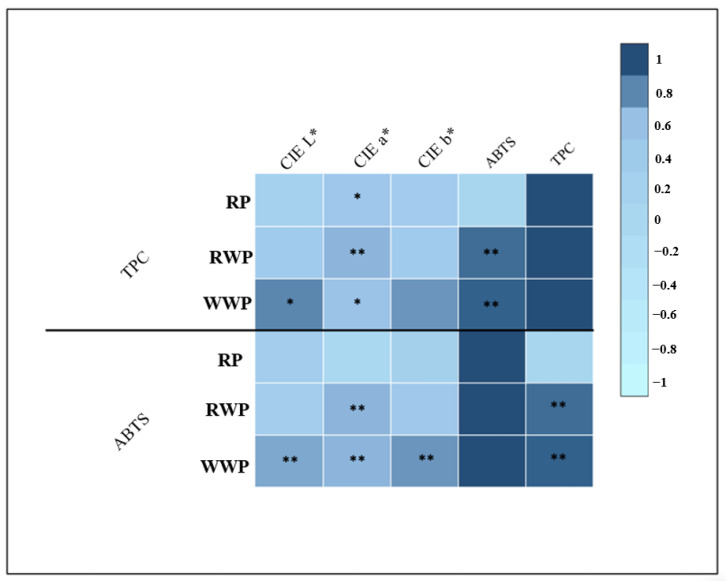
Heatmap of Pearson’s correlations based on measured parameters in RP, RWP, and WWP with different treatments. *: significant correlation at *p* < 0.05, **: significant correlation at *p* < 0.01.

Pearson’s correlation analysis, shown in Figure 2, reveals both positive and negative significant correlations between the different parameters assessed in this study.

In RP, RWP, and WWP significant bivariate correlations were found between instrumental color parameters, TPC, and ABTS. In RP, significant positive correlations between redness (CIE a*) and TPC were found (r = +0.438, *p* < 0.05), suggesting that the processing conditions that best preserved the red color also maintained higher levels of phenolic compounds. This may suggest that the carotenoids responsible for the red color of RP not only contribute to color intensity but may also exert a protective effect on phenolic compounds, helping to reduce their degradation under processing conditions. In this line, previous studies under similar or more intense conditions of HPTT have shown that phenolic compounds and antioxidant activity are well preserved in products with carotenoid compounds, like pumpkin [25,74] or carrot [63].

Regarding the redness (CIE a*) of RWP, also presented significant positive Pearson correlations with TPC (r = +0.637, *p* < 0.01) and ABTS (r = +0.639, *p* < 0.01). This is because malvidin and petunidin are anthocyanin pigments that typically exhibit blue-red hues [77]. Therefore, from a chromatic point of view, this change in phenolic compounds (anthocyanins) would modify the blue-red appearance of the processed RWP. Anthocyanins have a high antioxidant capacity, which explains the correlation between CIE a*, TPC, and ABTS. In fact, TPC showed highly significant correlations with antioxidant activity (r = +0.893, *p* < 0.001). This link indicates that the intensity of red not only reflects the amount of anthocyanins but also their contribution to the antioxidant bioactivity of the pomace.

Finally, in the WWP all instrumental color parameters (CIE L*, a*, and b*) presented significant positive correlations with TPC (r = +0.799, *p* < 0.001, for CIE L*) (r = +0.590, *p* < 0.01, for CIE a*), (r = +0.750, *p* < 0.001, for CIE b*). In addition, TPC and ABTS also presented strong positive correlations (r = +0.934, *p* < 0.001).

Given the strong correlations between CIE a* values and both TPC and ABTS in these by-products, optimizing processing conditions can be achieved efficiently, quickly, and cost-effectively by measuring redness (CIE a*) after processing.

## 4. Conclusions

RP (red pepper), RWP (red wine pomace), and WWP (white wine pomace) have demonstrated significant potential as food ingredients due to their high dietary fiber content, low-fat levels, and abundance of bioactive compounds with antioxidant properties. This study confirms that the bioactive compound content of these by-products is largely retained after high-pressure thermal treatment (HPTT). The effectiveness of HPTT is influenced by the specific type of by-product and the processing parameters applied. Thus, adapting the HPTT conditions to the characteristics of each by-product is essential to optimize the retention of quality attributes while ensuring food safety.

In RP, HPTT effectively preserved total phenolic content (TPC), total carotenoid content (TCC), antioxidant activity (ABTS), and key color parameters (CIE L* and a*), indicating a higher thermal stability of carotenoid-rich matrices. Additionally, HPTT may enable more effective microbial inactivation, suggesting the potential for extended shelf life; however, this should be further evaluated in future studies.

Conversely, RWP and WWP showed greater sensitivity to intense treatments, likely due to their higher phenolic content and the effects of adiabatic heating. In these cases, milder HPTT conditions—such as reduced pressure intensity, lower initial temperatures, or shorter holding times—may be more suitable, especially considering their naturally low water activity (a_w_) and acidic pH, which already contribute to microbiological stability.

On the other hand, a correlation between the color parameter CIE a* and the presence of bioactive compounds was identified, which opens the possibility of using color as a practical indicator to adjust and optimize industrial processes.

To consolidate and extend these findings, further studies are needed to provide relevant information on other key aspects of the HPTT, such as the microbiological evaluations to ensure the robustness of HPTT from a food safety perspective.

## Figures and Tables

**Table 1:** Experimental design.

Lot	Treatment Type	Pressure (MPa)	Time (min)	Temperature (°C)
1	Control	0.1	0	0
2	TT	0.1	5	65
3	TT	0.1	5	75
4	TT	0.1	5	85
5	HPTT	600	5	65 (83) *
6	HPTT	600	5	75 (93) *
7	HPTT	600	5	85 (103) *

TT: thermal treatment; HPTT: high-pressure thermal treatment. * Estimated final temperature considering adiabatic heating.

**Table 2:** Chemical composition of RP, RWP, and WWP.

	RP	RWP	WWP	*p*-Value
pH	5.18 a ± 0.00	3.48 b ± 0.01	3.31 c ± 0.01	***
a_w_	0.979 a ± 0.00	0.954 b ± 0.00	0.944 c ± 0.00	***
Moisture (g 100 g^−1^)	91.42 a ± 0.55	49.16 b ± 0.43	44.11 c ± 0.23	***
Fat (g 100 g^−1^)	0.59 c ± 0.18	3.26 a ± 0.02	2.33 b ± 0.03	***
Fiber (g 100 g^−1^)	1.94 c ± 0.21	38.14 a ± 0.29	34.46 b ± 0.22	***
Protein (g 100 g^−1^)	1.13 b ± 0.01	2.92 a ± 0.16	2.40 a ± 0.04	***

Different letters in the same row indicate statistical differences (Tukey-b’s test). *** *p* < 0.001.

## Data Availability

The original contributions presented in the study are included in the article. Further inquiries can be directed to the corresponding author.

## Abbreviations

The following abbreviations are used in this manuscript:

HPTT	High-pressure thermal treatment
HHP	Hydrostatic high pressure
TT	Thermal treatment
RP	Red pepper
RWP	Red wine pomace
WWP	White wine pomace
TPC	Total phenolic content
PPO	Polyphenol oxidase enzyme
a_w_	Water activity
TCC	Total carotenoid content
