# Correction: Sánchez-Ordóñez et al. Integral Valorisation of Agri-Food By-Products Through the Production of Food Ingredients Using High-Pressure Thermal Treatments. *Foods* 2025, *14*, 2214

**DOI:** 10.3390/foods14223957

**Published:** 2025-11-19

**Authors:** Miriam Sánchez-Ordóñez, Jorge A. Saraiva, Carlos A. Pinto, Jonathan Delgado-Adámez, M. Rosario Ramírez-Bernabé

**Affiliations:** 1 Centro de Investigaciones Científicas y Tecnológicas de Extremadura (CICYTEX), Instituto Tecnológico Agroalimentario (INTAEX), 06071 Badajoz, Spain; miriam.sanchezo@juntaex.es (M.S.-O.); jonathan.delgado@juntaex.es (J.D.-A.); 2 Laboratório Associado para a Química Verde/Associated Laboratory for Green Chemistry (LAQV) of the Network of Chemistry and Technology (REQUIMTE), Department of Chemistry, University of Aveiro, 3810-193 Aveiro, Portugal; jorgesaraiva@ua.pt (J.A.S.); carlospinto@ua.pt (C.A.P.)

## Error in Table

In the original publication [1], there was a mistake in Table 4 as published. The values in column *p* are not correct. A sentence should be eliminated (ns: Non-significant differences, *p* > 0.05). The corrected Table 4 appears below. 

In the original publication, there was a mistake in Table 5 as published. The values in column *p* are not correct. The sentences should be eliminated (** *p* < 0.01; ns: Non-significant differences, *p* > 0.05). The corrected Table 5 appears below. 

## Figures and Tables

**Table 4:** Instrumental color parameters, color changes (ΔE: control vs. treated), TPC (mg GAE 100 g^−1^ wet sample), and ABTS (μM TE ml^−1^ extract) of RWP with different treatments.

	Control	TT (5 min)			HPTT (600 MPa/5 min)			*p*
		65 °C	75 °C	85 °C	65 °C	75 °C	85 °C	
**Instrumental color**								
CIE L*	34.54 a ±1.68	33.62 ab ± 0.79	33.29 ab ± 0.33	32.60 ab ± 0.48	34.65 a ± 0.10	31.82 b ± 0.24	34.22 a ± 0.27	**
CIE a*	5.85 a ± 0.23	6.77 a ± 0.32	6.34 a ± 0.53	6.32 a ± 0.11	4.45 b ± 0.42	4.19 b ± 0.36	4.34 b ± 0.37	***
CIE b*	3.58 b ± 0.26	4.89 a ± 0.36	4.63 a ± 0.25	4.65 a ± 0.08	3.19 ab ± 0.12	3.35 ab ± 0.25	2.95 b ± 0.05	***
**Color changes**								
ΔE	-	1.91 ab ± 0.72	1.75 b ± 0.45	2.27 ab ± 0.46	1.46 b ± 0.41	3.20 a ± 0.36	1.68 b ± 0.37	***
**Nutritional molecules**								
TPC	107.78 a ± 1.32	93.98 abc ± 4.79	95.90 ab ± 4.30	79.62 cd ± 6.93	72.05 d ± 10.68	66.44 d ± 1.01	81.51 bcd ± 0.46	***
**Antioxidant activity**								
ABTS	1.30 a ± 0.01	1.18 ab ± 0.03	1.20 a ± 0.04	1.05 b ± 0.07	0.91 c ± 0.09	0.90 c ± 0.02	1.06 b ± 0.03	***

Values represent mean ± standard deviation. Different letters in the same row indicate statistical differences (Tukey-b’s test). ** *p* < 0.01 *** *p* < 0.001.

**Table 5:** Instrumental color parameters, color changes (ΔE: control vs. treated), TPC (mg GAE 100 g^−1^ wet sample), and ABTS (μM TE ml^−1^ extract) of WWP with different treatments.

	Control	TT (5 min)			HPTT (600 MPa/5 min)			*p*
		65 °C	75 °C	85 °C	65 °C	75 °C	85 °C	
**Instrumental color**								
CIE L*	38.88 abc ± 0.61	39.66 a ± 0.15	38.33 bcd ± 0.44	38.78 abc ± 0.18	39.19 ab ± 0.08	37.70 d ± 0.24	38.12 cd ± 0.18	***
CIE a*	8.78 a ± 0.19	8.78 a ± 0.08	8.62 a ± 0.06	8.39 a ± 0.34	6.49 b ± 0.37	7.20 b ± 0.23	7.34 b ± 0.51	***
CIE b*	12.40 a ± 0.33	12.92 a ± 0.03	11.84 ab ± 0.09	11.16 b ± 0.07	8.37 b ± 0.17	8.60 b ± 0.27	9.14 b ± 0.67	***
**Color changes**								
ΔE	-	0.94 b ± 0.11	0.82 b ± 0.37	1.32 b ± 0.18	4.66 a ± 0.11	4.29 a ± 0.3	3.66 a ± 0.78	***
**Nutritional molecules**								
TPC	205.59 a ± 1.95	200.77 ab ± 5.58	171.91 cd ± 11.84	186.26 bc ± 0.32	176.57 c ± 1.78	155.07 de ± 7.88	151.39 e ± 1.13	***
**Antioxidant activity**								
ABTS	2.39 a ± 0.07	2.30 abc ± 0.01	2.13 bc ± 0.19	2.36 ab ± 0.1	2.07 cd ± 0.03	1.83 de ± 0.04	1.78 e ± 0.05	***

Values represent mean ± standard deviation. Different letters in the same row indicate statistical differences (Tukey-b’s test). *** *p* < 0.001.
